# Supplementary material for: Isolation of the Thermostable β-Glucosidase-Secreting Strain Bacillus altitudinis JYY-02 and Its Application in the Production of Gardenia Blue
Source: Microbiol Spectr. 2022 Jul 14;10(4):e01535-22. doi: 10.1128/spectrum.01535-22 (PMC9431551; doi:10.1128/spectrum.01535-22)
Supplement: Supplemental file 1 — Supplemental material. Download spectrum.01535-22-s0001.pdf, PDF file, 1.4 MB [file spectrum.01535-22-s0001.pdf]

SUPPLEMENTARY MATERIALS

"Isolation of *Bacillus altitudinis* JYY-02 secreting a thermal-stable β-glucosidase and its application in production of gardenia blue"

Table 1S Physiological and biochemical experiments of *B. altitudinis* JYY-02

Strain	*E. coli*	*Bacillus subtilis*	*Bacillus altitudinis .JYY-02*
V.P	－	＋	＋
Methyl Red	＋	－	－
Citrate	－	＋	＋
Glucose fermentation	－	＋	＋
Lactose fermentation	－	＋	＋
Sucrose fermentation	＋	－	＋
Lead Sulfide	－	－	＋
Amylase	－	＋	－
Lipase	－	＋	＋

＋ indicates positive, － indicates negative.

Figure 1S

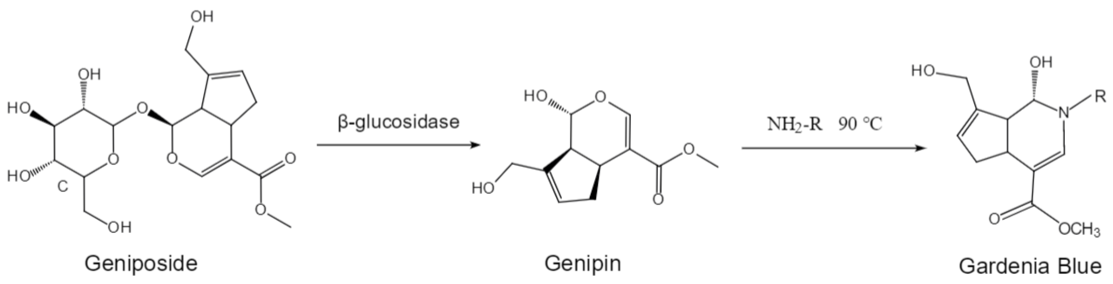

Figure 1S Production process of gardenia blue.

Figure 2S

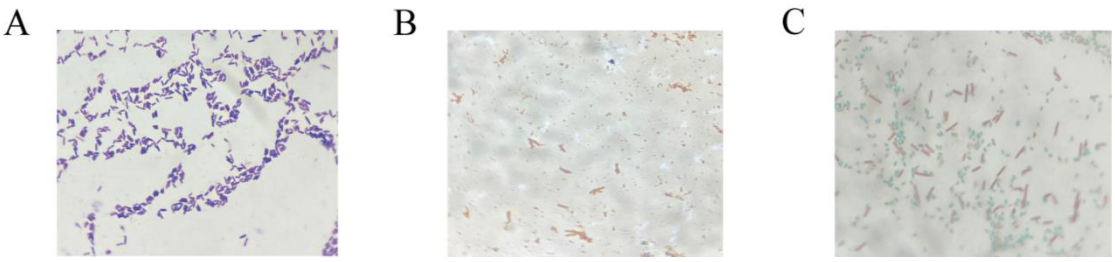

Figure 2S Morphological characters of *B. altitudinis* JYY-02. A, Gram staining of *B. altitudinis* JYY-02. B, Flagellar staining of *B. altitudinis* JYY-02. C, *Bacillus* staining of *B. altitudinis* JYY-02.

Figure 3S

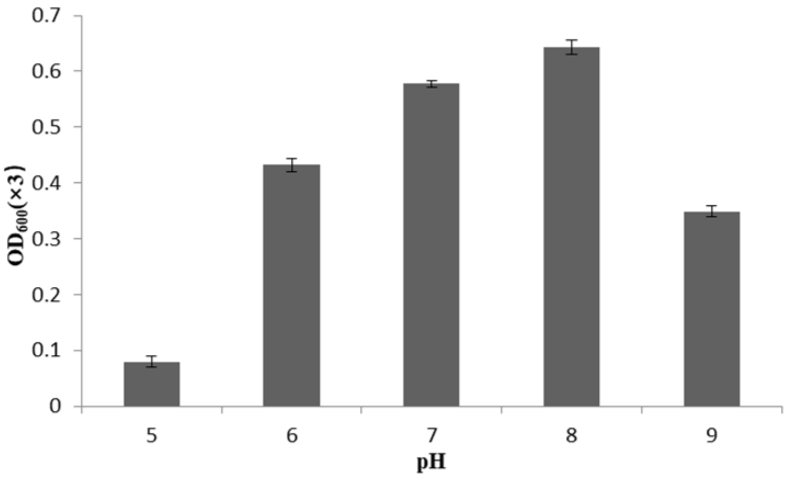

Figure 3S Effect of different pH values on the growth of *B. altitudinis* JYY-02.

Figure 4S

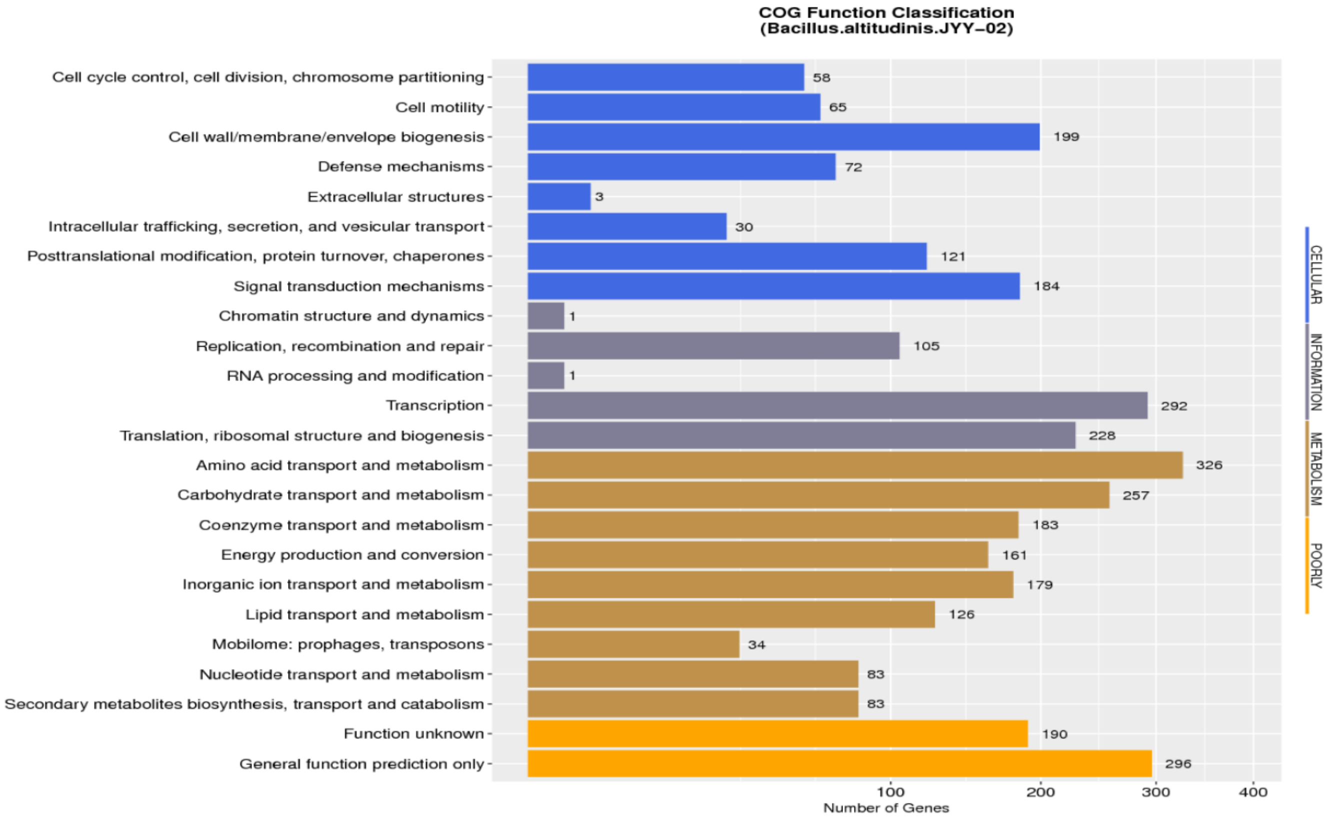

Figure 4S COG database function annotation distribution chart.

Figure 5S

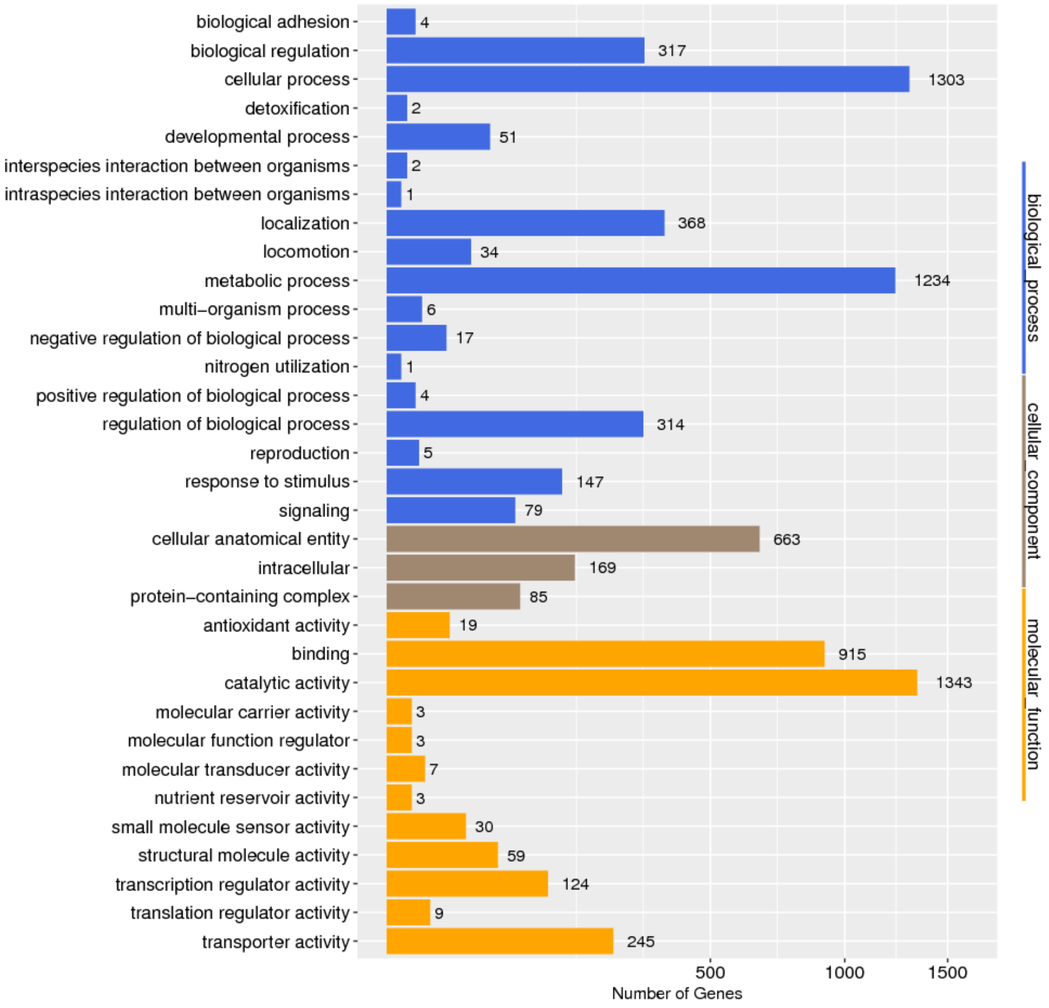

Figure 5S GO database function annotation distribution chart of the genome from *B. altitudinis* JYY-02.

Figure 6S

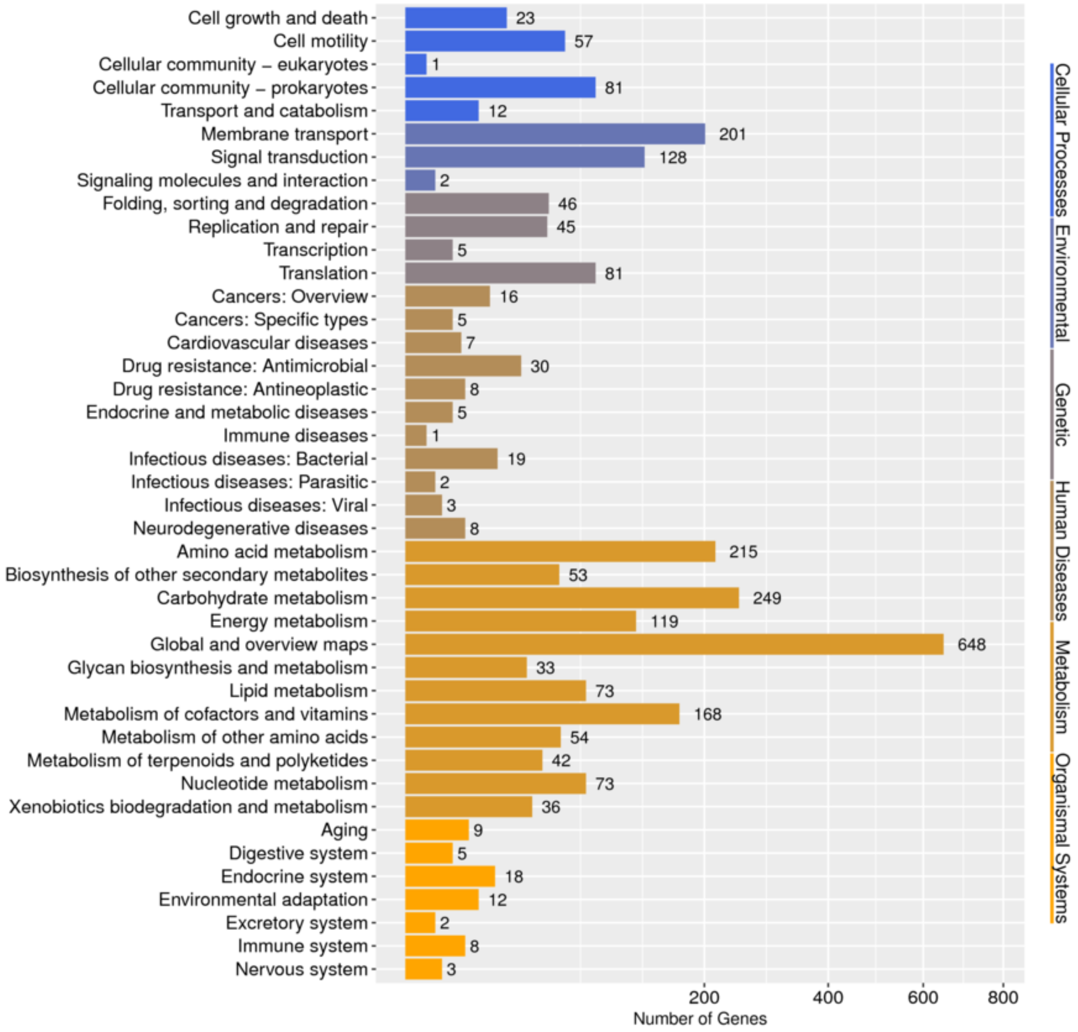

Figure 6S KEGG database function annotation distribution chart of the genome from *B. altitudinis* JYY-02.

Figure 7S

```
1      ATGACGACAATAAAAGGTTTTCCAAAAGGATTTTTATGGGGCGGCGCCATTGCCGCCAACCAAGCAGAAGGCGCTTGGAATGTAGATGGA
1       M  T  T  I  K  G  F  P  K  G  F  L  W  G  G  A  I  A  A  N  Q  A  E  G  A  W  N  V  D  G
91     AAGGGCCCATCTGTTGCTGATATTGCGATGTACCGGTCTAATTTGAGTGTAGAAGATTATGAAGGGCATCTAGCTGTTACTTCTGAAAAT
31      K  G  P  S  V  A  D  I  A  M  Y  R  S  N  L  S  V  E  D  Y  E  G  H  L  A  V  T  S  E  N
181    ATAGATCGAGCGATGAAAGACCCAGATGATAAAAAGTATCCAAAACGCAGAGGTGTTGATTTCTATCATCACTATAAAGAGGATTTGGCT
61      I  D  R  A  M  K  D  P  D  D  K  K  Y  P  K  R  R  G  V  D  F  Y  H  H  Y  K  E  D  L  A
271    CTATTTGCTGAAATGGGCTTTAAAACTCTTCGCATCTCTATTGCATGGAGCCGTATCTTCCCAACTGGAGAAGAGAACGAACCAAATGAA
91      L  F  A  E  M  G  F  K  T  L  R  I  S  I  A  W  S  R  I  F  P  T  G  E  E  N  E  P  N  E
361    AAAGGACTCCAATTTTACGATCGTTTATTTGCAGAAATGAAAAAACACAACATAGAACCGATTGTGACACTGTCACATTATGAAATGCCG
121     K  G  L  Q  F  Y  D  R  L  F  A  E  M  K  K  H  N  I  E  P  I  V  T  L  S  H  Y  E  M  P
451    TTAGCACTGAGCGTGAAATATAATGGCTGGGTTGAAAGAAAAGTCGTTGATCTCTTTGTTAAGTTCGCCAATGTATGTTTTGAACGCTAT
151     L  A  L  S  V  K  Y  N  G  W  V  E  R  K  V  V  D  L  F  V  K  F  A  N  V  C  F  E  R  Y
541    AAACATGATGTGAAATATTGGCTGACCTTTAATGAAATAGATAGTATTCACCGCCACTCTTTTATTACAGCAGGAATTATTCCTGATCGC
181     K  H  D  V  K  Y  W  L  T  F  N  E  I  D  S  I  H  R  H  S  F  I  T  A  G  I  I  P  D  R
631    TGTCCAGAAGGGAAAGAGGAAGAGACGGTTTATCAAGCACTCCATCATCAATTCATTGCCTCTGCACTCGTAACAGCAGATTGTCATCGC
211     C  P  E  G  K  E  E  E  T  V  Y  Q  A  L  H  H  Q  F  I  A  S  A  L  V  T  A  D  C  H  R
721    ATCATACCAGGCAGTCAAGTAGGGTGTATGCTGACAAAATTAACAACGTATCCGCACACTTGTCATCCGAATGATGTGGAGCGGGCATTG
241     I  I  P  G  S  Q  V  G  C  M  L  T  K  L  T  T  Y  P  H  T  C  H  P  N  D  V  E  R  A  L
811    AAACAAAACTTAGAAAACTATTTCTATGCAGATGTTCAGGTATTTGGTGAATACCCGCCGCTTATCAAGCGCATGCTTGAGAGAAAAAAC
271     K  Q  N  L  E  N  Y  F  Y  A  D  V  Q  V  F  G  E  Y  P  P  L  I  K  R  M  L  E  R  K  N
901    ATTCATATCCAAATGGAATCAGATGATCTTTCTATTTTAAAAGAAAATACAGTAGATTTTATCTCGTTCAGCTACTATATGTCTTTAACT
301     I  H  I  Q  M  E  S  D  D  L  S  I  L  K  E  N  T  V  D  F  I  S  F  S  Y  Y  M  S  L  T
991    GAATCAGCTGATGAAGGATTAGAAAAAACGGATGGAAATACCATCCGTGGGGTCAAAAATCCTTACCTTCCATCTACTGATTGGGGATGG
331     E  S  A  D  E  G  L  E  K  T  D  G  N  T  I  R  G  V  K  N  P  Y  L  P  S  T  D  W  G  W
1081   CAAATTGATCCGGTTGGTCTGAAAATTTCTTTAATAGAATTATATGATCGTTATCAAAAACCACTGATCATTGTAGAAAACGGCATGGGT
361     Q  I  D  P  V  G  L  K  I  S  L  I  E  L  Y  D  R  Y  Q  K  P  L  I  I  V  E  N  G  M  G
1171   GCAAAAGATGTTGTTGAAAGTGATGGATCAATTCACGATGATTATCGTATTAACTATTTCAAAGAGCATTTCCGCCAAATGAGAGAAGCG
391     A  K  D  V  V  E  S  D  G  S  I  H  D  D  Y  R  I  N  Y  F  K  E  H  F  R  Q  M  R  E  A
1261   GTGGAAGAAGGCGTTGACCTCTTCGGATATACGAGCTGGGGCTCAATTGATATTATTAGTGCAGGTACATCACAGATGTCAAAACGATAC
421     V  E  E  G  V  D  L  F  G  Y  T  S  W  G  S  I  D  I  I  S  A  G  T  S  Q  M  S  K  R  Y
1351   GGGTTCATTCATGTAGATCAAGATGACGATGGAAACGGTACATTAAAACGTTCTCGTAAAGACTCATTTTATTGGTACAAAAAGGTCATT
451     G  F  I  H  V  D  Q  D  D  D  G  N  G  T  L  K  R  S  R  K  D  S  F  Y  W  Y  K  K  V  I
1441   GAAACAAATGGTGAATCGCTTGATTGA
481     E  T  N  G  E  S  L  D  *
```

Figure 7S Nucleotide sequence of the β-glucosidase gene and the amino acid sequence encoded by β-glucosidase gene.

Figure 8S

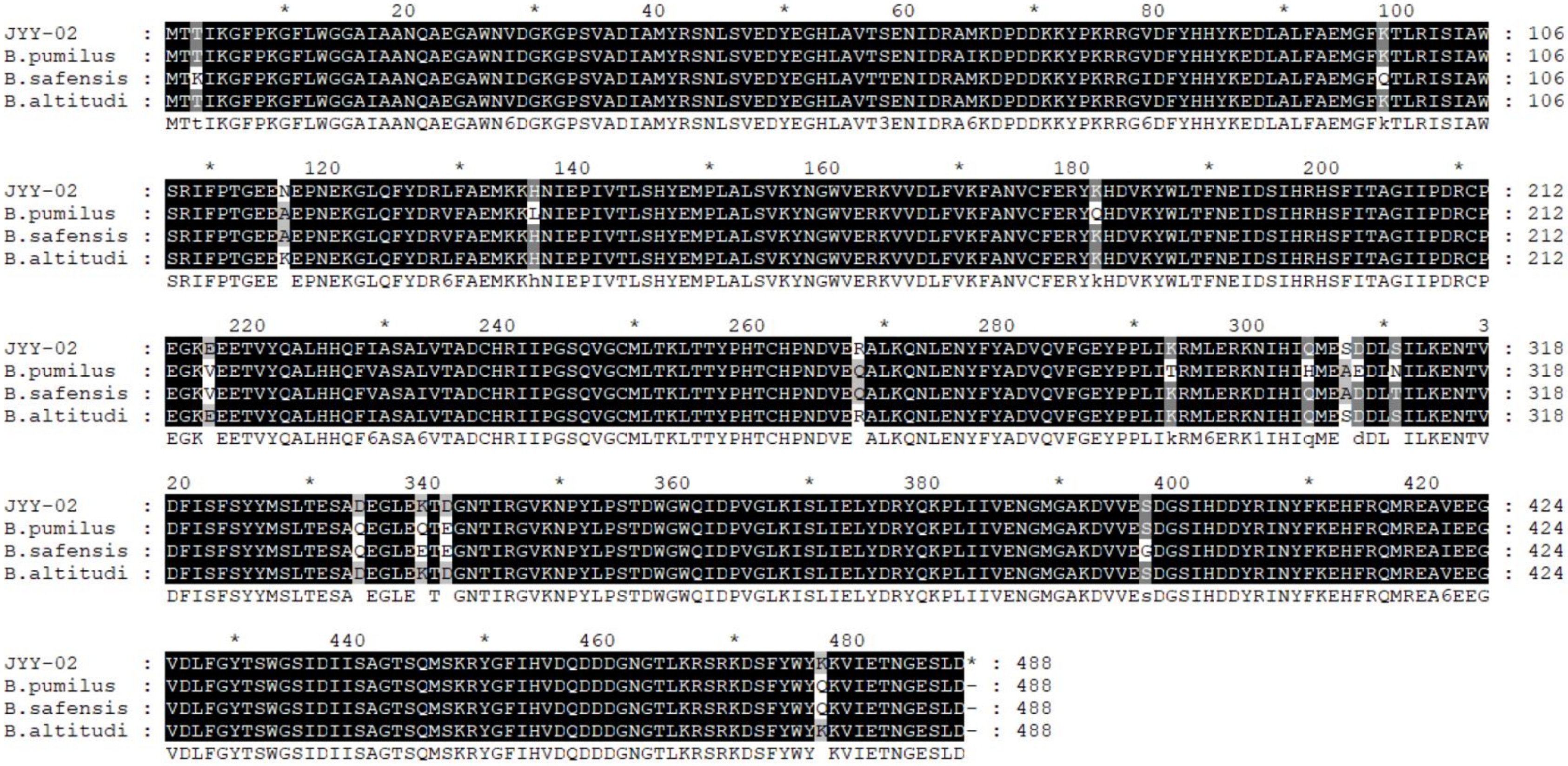

Figure 8S Comparison of the amino acid sequence of the β-glucosidase of *B. altitudinis* JYY-02 with the homologous β-glucosidase of other species.
